# Preclinical evaluation of *Lactiplantibacillus plantarum* as a probiotic alternative against multidrug-resistant avian pathogenic *Escherichia coli* in chickens

**DOI:** 10.3389/fimmu.2025.1640600

**Published:** 2025-11-04

**Authors:** Hanem El-Sharkawy, Ahmed Mohamed Abdelsalam, Mohamed Marzok, Yamen Hegazy, Hussein Babiker, Amin Tahoun

**Affiliations:** ^1^ Department of Poultry and Rabbit Diseases, Faculty of Veterinary Medicine, Kafrelsheikh University, Kafrelsheikh, Egypt; ^2^ Department of Microbiology, Immunology and Molecular Genetics, University of Texas Health Science Center at San Antonio, San Antonio, TX, United States; ^3^ Department of Clinical Sciences, College of Veterinary Medicine, King Faisal University, Al-Ahsa, Saudi Arabia; ^4^ Department of Clinical Veterinary Medical Sciences, Faculty of Veterinary Medicine, Irbid, Jordan

**Keywords:** broilers, APEC, antimicrobial, *Lactobacillus plantarum*, macrophage, colonization

## Abstract

**Objectives:**

Avian pathogenic *Escherichia coli* (APEC) is a leading cause of disease and mortality in broiler chickens, resulting in substantial economic losses. Probiotics such as *Lactiplantibacillus plantarum* (*L. plantarum*) have shown potential to enhance host immunity and limit pathogen colonization, but their efficacy against APEC is not fully understood.

**Methods:**

One hundred diseased broilers from 20 farms were screened for *E. coli* isolation, serotyping, and antimicrobial resistance. The probiotic strain *L. plantarum* ATS1 (GenBank accession no. PV478081.1), previously isolated and partially characterized in our laboratory, was evaluated for adhesion to Caco-2 epithelial cells, survival in murine macrophages, and *In Vivo* effects on growth performance, serum IgY levels, and protection against oral challenge with APEC O126:K71 at 28 days of age.

**Results:**

Twenty-three *E. coli* isolates were recovered, with (APEC) predominating (80%) and 20% identified as Shiga toxin-producing O26 strains. Multidrug resistance was common, including complete resistance to cefixime and aztreonam. *L. plantarum* showed strong adhesion to epithelial cells (5.65 ± 1 bacteria/cell) and survived within macrophages. *In Vivo* supplementation increased serum IgY, improved body weight gain and feed conversion ratio, reduced cecal and hepatic APEC colonization, and lowered mortality following challenge.

**Conclusions:**

*L. plantarum* ATS1 provides protective and immunomodulatory effects against APEC by supporting intestinal colonization, surviving in macrophages, and enhancing humoral immunity. These findings highlight its potential as a probiotic strategy to improve broiler health and reduce dependence on antibiotics.

## Introduction

*Escherichia coli* (*E. coli*) remains a prominent pathogen in poultry production systems, where it is a primary cause of colibacillosis, an infectious disease responsible for significant economic losses due to elevated mortality, reduced growth performance, and the rejection of carcasses during processing (1, 2). Among its various strains, avian pathogenic *E. coli* (APEC) is associated with multiple clinical conditions in poultry, including omphalitis, septicemia, cellulitis, yolk sac infections, and respiratory disease. APEC strains are genetically related to human extraintestinal pathogenic *E. coli* (ExPEC), raising concerns about their zoonotic potential (3–5).

There is growing evidence of the emergence of multidrug-resistant (MDR) APEC isolates, primarily attributed to the extensive and often inappropriate use of antibiotics in poultry, both for disease prevention and treatment (6, 7). This trend not only compromises treatment efficacy but also poses public health risks due to the potential transfer of resistance genes through the food supply chain (8, 9).

In Egypt, the most commonly used antibiotics on poultry farms cephalosporins, quinolones, tetracyclines, and trimethoprim-sulfamethoxazole are also the most frequently resisted by APEC (10–12).

In response to this global challenge, the use of probiotics has attracted increasing interest as a sustainable and safe alternative to conventional antibiotics in poultry production. Numerous studies have highlighted the beneficial effects of *Lactobacillus* species, including their roles in immune modulation, pathogen exclusion, and maintenance of gut integrity (13, 14). *Lactiplantibacillus plantarum* (*L. plantarum*) has demonstrated notable probiotic potential by reducing the colonization of intestinal pathogens and enhancing host immune responses (15, 16). *L. plantarum* is known to produce antimicrobial metabolites, including bacteriocins and short-chain fatty acids, which have the capacity to inhibit pathogenic bacteria (17, 18).

The *L. plantarum* postbiotic demonstrated protective effects against *Salmonella* infection in broilers, likely by inhibiting NLRP3 inflammasome activation and promoting a healthier gut microbiota balance (19, 20). Dietary supplementation with *L. plantarum* enhanced growth performance and health status in broiler chickens, potentially through modulation of the gut microbiota composition favoring short-chain fatty acid (SCFA)-producing bacteria. These findings indicate that *L. plantarum* may represent a promising alternative to antibiotic growth promoters in poultry production (21–23).

Interestingly, some probiotic strains, including *L. plantarum*, have shown the ability to persist within macrophages, which may contribute to prolonged immune interaction and stimulation (24). Despite this, the underlying mechanisms by which *L. plantarum* survives intracellularly and influences autophagy and host immunity in poultry are still not fully understood.

The present study was conducted to determine the prevalence and antimicrobial resistance patterns of APEC in broiler farms in Egypt and to assess the probiotic efficacy of *L. plantarum*, focusing on its ability to adhere to epithelial cells, survive in macrophages, and provide protective effects against APEC infection *In Vivo.*

## Materials and methods

### Samples bacteriological assay

One hundred chickens from 20 broiler farms with respiratory and septicemic signs in Kafr El-Sheikh Province were sampled from February to August 2024. Diseased chickens were humanely sacrificed, and internal organ samples were collected aseptically for bacteriological examination. The samples were inoculated into tryptone soy broth (Oxoid, UK) and incubated overnight at 37°C. Cultures were streaked onto eosin methylene blue (EMB) agar (Oxoid, UK) for selective *E. coli* isolation, followed by biochemical identification via the API 20E system (BioMérieux, France).

### Serological identification of *E. coli*

Twenty-three *E. coli* isolates from chickens exhibiting airsacculitis, pericarditis, and perihepatitis from five broiler farms were serologically identified following the methods of Kok et al., (25) (25) at the reference laboratory of the animal health research institute of the Agricultural Research Centre, Dokki, Cairo, Egypt.

### Antimicrobial susceptibility testing

Antimicrobial susceptibility of the *E. coli* isolates was determined by the Kirby–Bauer disc diffusion method on Mueller–Hinton agar (26), and inhibition zone diameters were interpreted according to CLSI guidelines (27). The antibiotic discs tested (Oxoid, UK) were chloramphenicol (30 µg), sulfamethoxazole–trimethoprim (25 µg), tetracycline (30 µg), gentamicin (10 µg), streptomycin (10 µg), colistin sulphate (10 µg), cefoperazone (75 µg), cefepime (30 µg), cefixime (5 µg), aztreonam (30 µg), ceftazidime (30 µg), cefotaxime (30 µg), imipenem (10 µg), and meropenem (10 µg). The numbers of isolates classified as sensitive, intermediate, or resistant to each antibiotic are presented in **Table 1**.

**Table 1:** Distribution of *E. coli* isolates (n = 23) recovered from broiler chickens according to organ source.

Organ source	Number of isolates	Percentage of total (%)
Liver	8	34.8%
heart	6	26.1%
Lung	4	17.4%
Gallbladder	3	13.0%
Air sacs	2	8.7%
Total	23	100%

### Minimal inhibitory concentration

Colistin-resistant F strains were further assessed via the E-strip method on Mueller–Hinton agar. The plates were incubated for 16–18 hours before MIC determination.

### *Lactiplantibacillus plantarum* strain and preparation

The probiotic strain used was *L. plantarum* ATS1 (GenBank accession no. PV478081.1), previously isolated and characterized in our laboratory. The strain was grown anaerobically in de Man–Rogosa–Sharpe (MRS) broth at 37°C overnight, centrifuged, washed in PBS, and resuspended in DMEM (OD_660_ = 0.7) for *in vitro* assays or diluted in PBS for oral supplementation.

### Adherence assay

Briefly, stationary-phase *L. plantarum* cultures grown in MRS broth were diluted 1:10 in PBS. An aliquot of this culture was used to challenge a confluent Caco-2 monolayer at a multiplicity of infection (MOI) of ~100 (bacteria to epithelial cells) in triplicate at 37°C with 5% CO_2_ for 2 hours.

For direct visualization of adherent bacteria, the cells were fixed in methanol for 5 minutes at room temperature and stained with Giemsa stain. A total of 100 cells were examined under a light microscope, and the number of adherent bacteria per cell was counted in randomly selected fields.

### Macrophage phagocytosis and *L. plantarum* survival and growth within macrophages assays

264.7 murine macrophages were used to assess bacterial phagocytosis via gentamicin protection assays (28). *L. plantarum* strain ATS1 cultures grown in MRS broth were incubated anaerobically at 37°C overnight. The bacterial culture was centrifuged, washed twice with PBS, and resuspended in DMEM to an OD_660_ of 0.7. This suspension was used to infect a confluent Raw 264.7 macrophage monolayer at an MOI of ~100 in triplicate.

To examine *L. plantarum* survival and growth within macrophages, the bacteria were kept with the cells for time point challenge. After 30 min of infection, the cells were washed three times with PBS, and DMEM containing 250 μg/mL gentamicin was added. The cells were incubated at 37°C for the indicated time points before analysis via microscopy.

A total of 100 cells were examined under a light microscope, and the number of internalized bacteria per cell was counted in randomly selected fields. Replicate samples were used for immuno-flourcent analysis.

### Staining the cells for light microscope

For visualization of internalized bacteria, macrophages were fixed in 100% cold methanol for 8 minutes at room temperature and stained with Giemsa stain (Muto Pure Chemicals, Tokyo, Japan) per the manufacturer’s instructions. Bacteria per macrophage were counted in randomly selected fields.

### Fluorescence microscopy

Following *L. plantarum* challenge, Raw 264.7 macrophages were processed for fluorescence microscopy. Infected cells were washed with PBS, fixed in 4% paraformaldehyde (pH 7.2), and permeabilized with 100% cold methanol for 8 minutes at room temperature. The cells were stained with primary mouse anti-LAMP-1 (Abcam) and detected with Alexa Fluor 568-conjugated goat anti-mouse IgG (Molecular Probes). Nuclei were stained with DAPI (Molecular Probes), washed, and mounted in ProLong Gold antifade mounting medium.

### Live/died bacterial staining

After the bacterial challenge, the macrophages were washed with PBS and incubated with gentamicin to eliminate extracellular bacteria. The cells were stained via the live/dead bacLight bacterial viability Kit (Molecular Probes) according to the manufacturer’s protocol. After staining, the coverslips were mounted and sealed with clear nail polish, and images were acquired via a Zeiss Axiovert confocal microscope.

### Ethical approval

All animal experiments were carried out in accordance with institutional guidelines and were approved by the Institutional Animal Care and Use Committee of the Faculty of Veterinary Medicine, Kafrelsheikh University, Egypt (Approval No. KFS-IACUC255/2025).

### Experimental infection

Forty Cobb500 broiler chicks, aged one day, were randomly distributed into four treatment groups (10 birds each), with each group housed separately and provided free access to water and antibiotic free feed.

The groups were as follows: Group 1 (Control): Received only the standard basal diet throughout the study. Group 2 *(L. plantarum*): Received the basal diet and daily oral supplementation with *Lactobacillus plantarum* (1 × 10^9^ CFU/mL) from day 1 onward. Group 3 (APEC): Fed the basal diet and challenged orally with APEC O126:K71 at 28 days of age using a dose of 1 × 10^8^ CFU/mL. Group 4 (*L. plantarum* + APEC): Supplemented with *L. plantarum* from day 1 and challenged with APEC at the same dose and time point as Group 3. The APEC strain used was nalidixic acid-resistant and known to cause systemic infection. The challenge dose (1 × 10^8^ CFU/mL) was selected based on established models that demonstrate the ability of APEC to cause septicemia and lesions such as airsacculitis, pericarditis, and hepatic inflammation, thereby validating the use of this dose to assess probiotic efficacy under disease pressure. All birds were closely monitored for clinical signs following inoculation, and necropsy was performed to assess organ pathology and confirm bacterial presence in target tissues.

### Bacterial challenge and enumeration

Cecal and liver samples (1 g) were suspended in 9 mL of PBS and serially diluted. Aliquots (100 μL) were spread onto MacConkey agar and incubated at 37°C for 16 hours. Colonies were counted to determine APEC CFU/g.

### Measurement of the total serum IgY level

At two weeks after the challenge, blood was collected from all experimental birds (n = 40; 10 per group). About 2 mL was taken from the wing vein of each bird, and sera were separated by centrifugation at 3,000 rpm for 10 minutes. For ELISA (ZeptoMetrix Corporation), 100 µL of each serum sample was added to the coated wells in duplicate together with a standard curve (7.8–125 ng/mL). After incubation and washing, wells were treated with HRP-labeled anti-chicken IgY, followed by substrate solution. The reaction was stopped, and absorbance was measured at 450 nm using a Varioskan Lux reader (Thermo Fisher, Germany). IgY concentrations were calculated from the standard curve.

### Statistical analysis

Statistical analyses were performed using SPSS v23.0. Two-way repeated-measures ANOVA assessed differences in feed conversion ratio and weight gain among control, *L. plantarum*, APEC, and *L. plantarum* + APEC groups over time, with Tukey’s *post hoc* test for pairwise comparisons (P < 0.05). One-way ANOVA was used to compare total serum IgY, fecal bacterial counts (log CFU), and RAW 264.7 macrophage responses to *L. plantarum* under time-course experiments with autophagy inhibitors (29). Differences between APEC and APEC + *L. plantarum* groups were evaluated using two-tailed Student’s t-tests (P < 0.05).

## Results

### Adherence of *L. plantarum* to caco-2 epithelial cells

**Figure 1:**
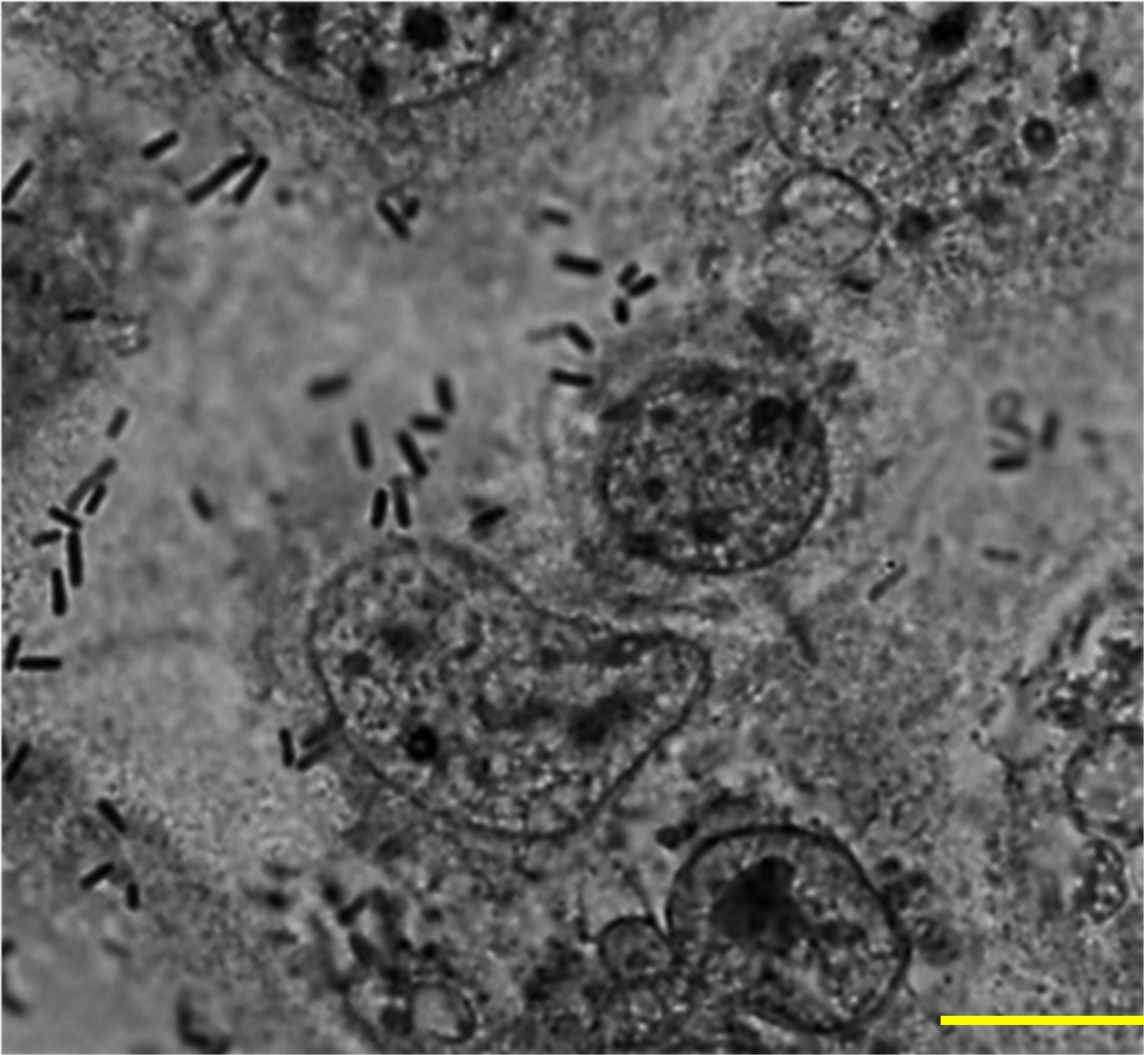
Adherence of L. plantarum to Caco-2 epithelial cells. A subconfluent monolayer of Caco-2 epithelial cells were challenged with *L. plantarum* at a multiplicity of infection of 1:100 at 37°C, 5% CO2 for 2 h and epithelial cells were stained with Giemsa stain. A total of 100 cells were examined under a light microscope, and the number of bacteria adhering to each cell was counted in 20 randomly selected fields. The number of attached *L. plantarum* per Caco-2 cell was 5.65 ± 1.18. Images were acquired via phase contrast microscopy (BX-43, Olympus Co., Tokyo, Japan). Scale bar, 10 µm.

*L. plantarum* exhibited characteristic compact and diffuse adherence to Caco-2 epithelial cells (**Figure 1**). Quantitative analysis revealed an average bacterial attachment of 5.65 ± 1.18 bacteria per epithelial cell. *L. plantarum* activates vacuolation and grows in murine macrophages.

**Figure 2:**
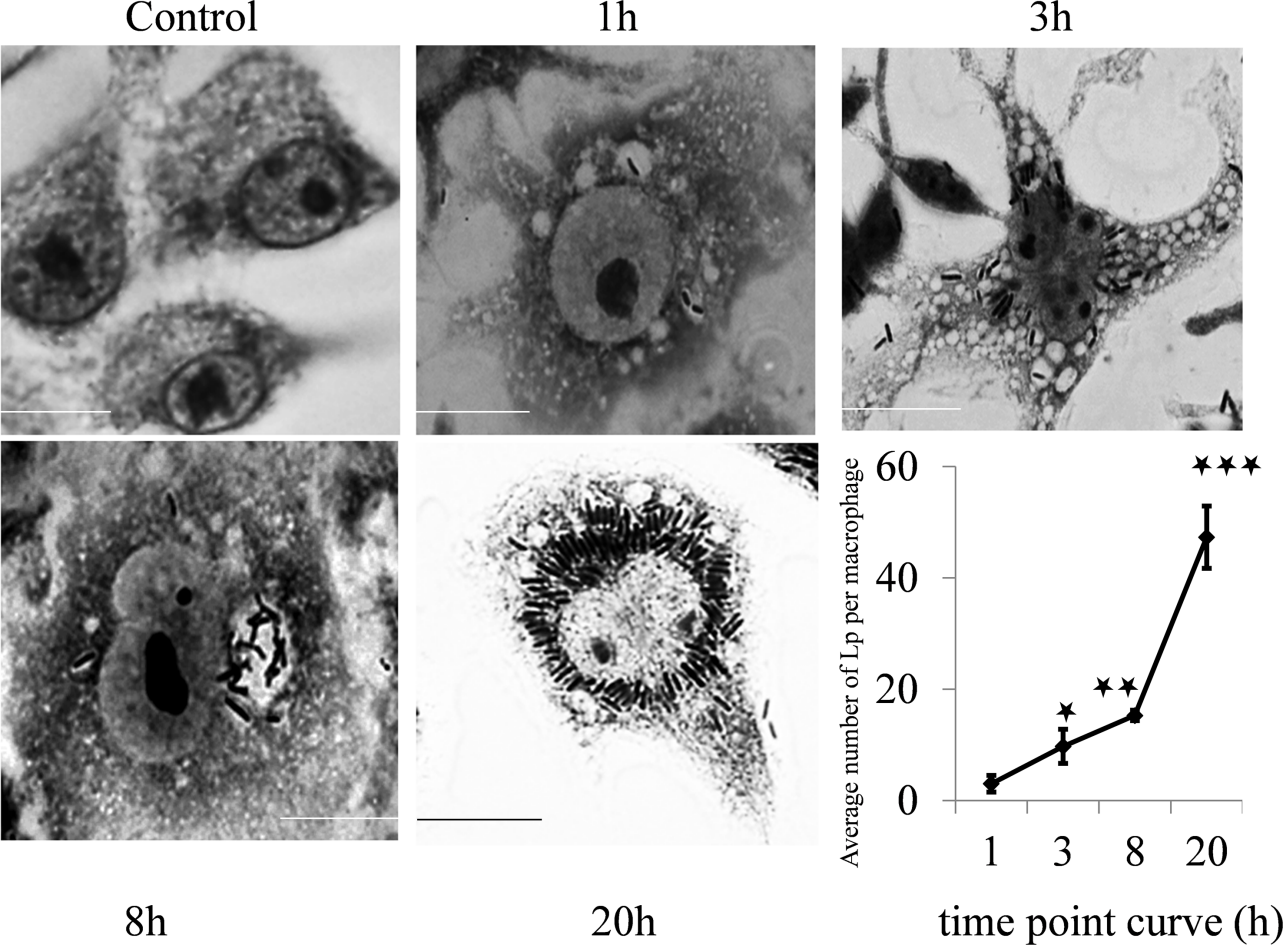
Time point interaction of *L. plantarum* with murine macrophages. Raw 264.7 macrophages were challenged with the *L. plantarum* strain at a panel of time points and stained with Giemsa stain. The control cells did not exhibit vacuolation, but vacuolation of the cell cytoplasm increased with time from 1 h to 20 h post challenge with *L. plantarum*. The bacteria were able to multiply and survive in vacuoles (autophagosomes), and the number of formed compact microcolonies markedly increased in the macrophages (p<0.001). Scale bar, 10 μm. *p < 0.05; **p < 0.01; ***p < 0.001.

To assess whether autophagy influences the interaction of *L. plantarum* with macrophages, a gentamicin protection assay was performed. *L. plantarum* forms characteristic compact vacuoles, presumably phagosomes, within macrophages. Within these vacuoles, bacteria were able to proliferate, leading to the identification of the *L. plantarum*-containing vacuole (LpCV) (**Figure 2**).

Quantitative analysis revealed a significant increase in the *L. plantarum* bacterial load over time. The mean bacterial count per macrophage was 3.05 ± 1.50 at 1 hour post infection, increasing to 9.75 ± 3.05 at 3 hours (p < 0.001). The bacterial count further increased significantly at 8 hours (15.3 ± 0.92, p < 0.001) and peaked at 20 hours (47 ± 5.58, p < 0.001) (time point curve, **Figure 2**). These results indicate that *L. plantarum* effectively survives and proliferates within murine macrophages, suggesting a role for autophagy in bacterial survival.

### Identification of *L. plantarum*-containing vacuole

**Figure 3:**
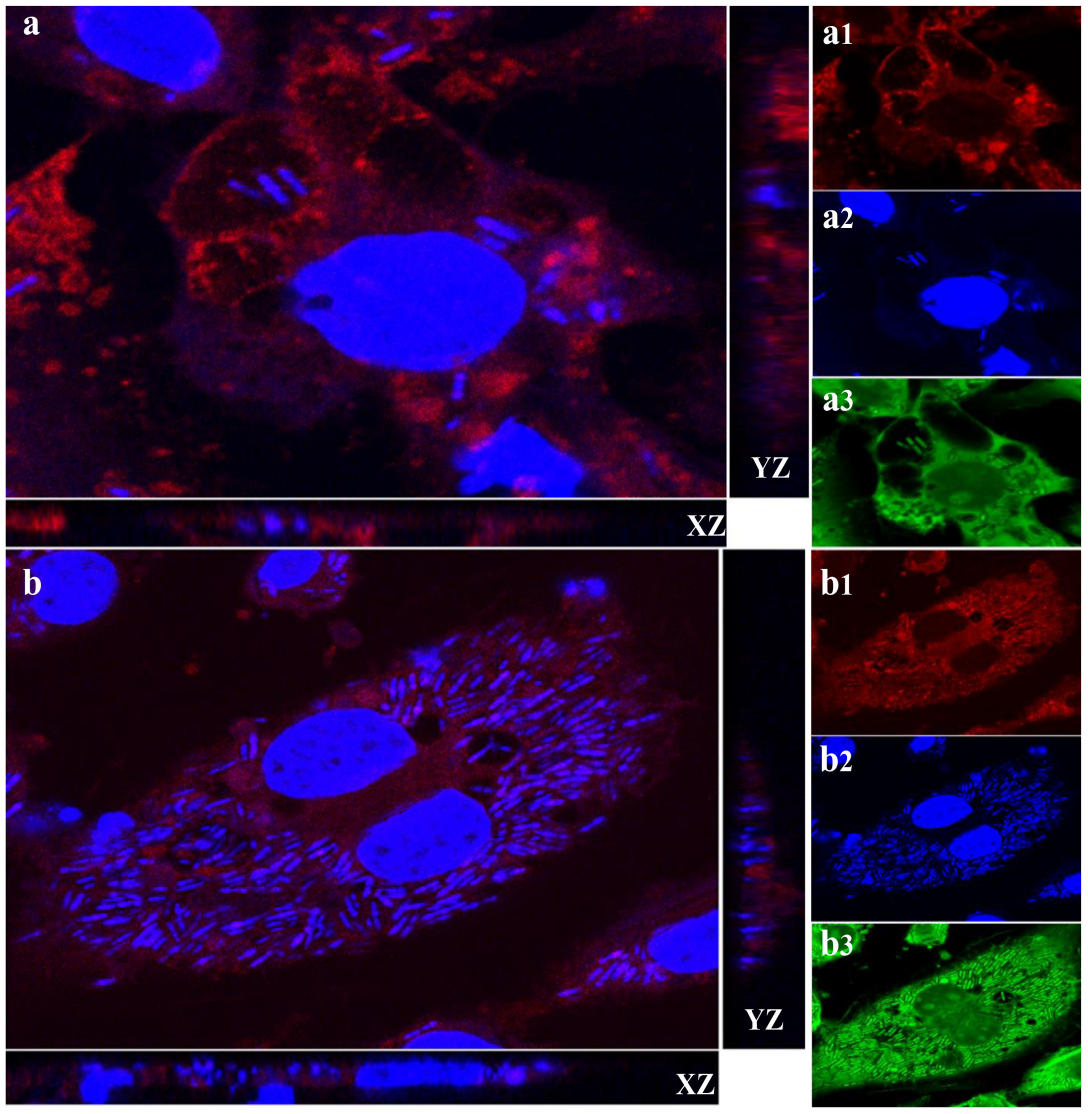
*Lactobacillus plantarum* is able to multiply in macrophages via LAMP-1. A subconfluent monolayer of raw 264.7 murine macrophages was challenged with *Lactobacillus plantarum* for 4 hours **(a)** and 20 hours **(b)** at 37°C and 5% CO_2_ for 4 hours and 20 hours. The cells were fixed in 4% (v/v) PFA (pH 7.2) and permeabilized with 100% (v/v) cold methanol. The cells were stained for LAMP-1 red (**a1** and **b1**), and the nuclei and bacteria were stained blue (**a2** and **b2**) or green of Live/dead stain (**a3** and **b3**). Images were acquired using a Zeiss Axiovert, objective x63. Orthogonal x–z and y–z sections of fluorescent images show internalized bacteria. The results are representative of three independent experiments. The number of *L. plantarum* (blue and green) increased, and Lactobacillus plantarum was localized to LAMP-1. Scale bar, 10 μm.

**Figure 4:**
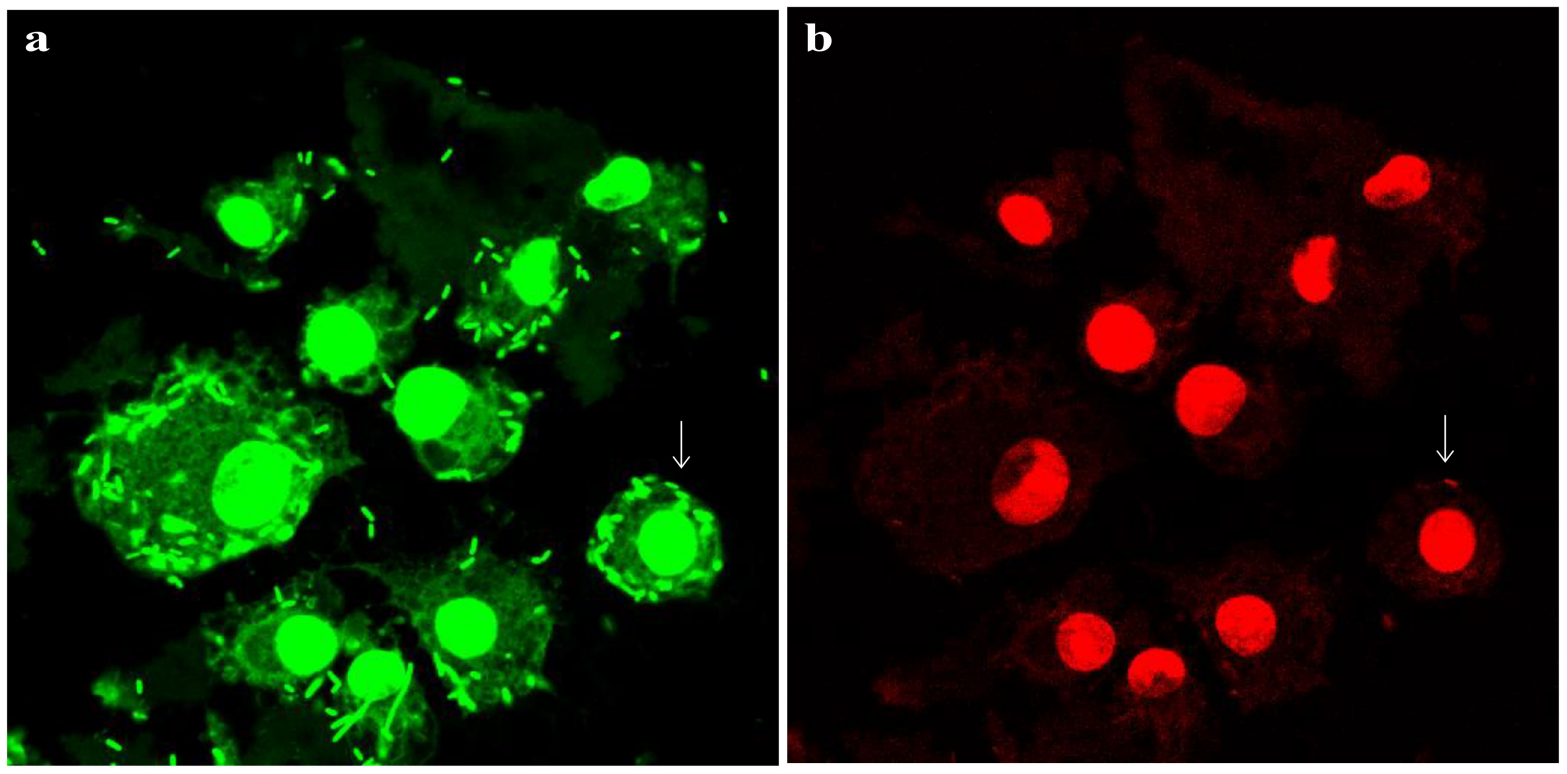
Survival of *L. plantarum* in raw 264.7 macrophages. Live/dead staining of *L. plantarum*-challenged murine macrophages for 20 h. Bacteria were added to the cells at a reduced MOI of 40:1 compared with the time point and inhibitor data (100:1). All bacteria should stain green **(a)**, while bacteria that have lost cell wall/membrane integrity should also stain red **(b)**. Arrows show examples of individual dead bacteria. Pictures were acquired via a Zeiss Axiovert confocal system (x 63 objective) at optimal z-slice sampling rates, as determined via Zeiss software, with a 1024 × 1024-pixel image size. Scale bar, 10 µm.

Given the vacuolation observed in macrophages exposed to *L. plantarum*, we considered that autophagy might help bacterial persistence by limiting lysosomal degradation. Over time, an increasing number of bacteria were detected within LAMP-1–positive vacuoles, and replication of *L. plantarum* was evident (**Figure 3**). To further confirm bacterial viability, macrophages were challenged with *L. plantarum* at a reduced MOI (40:1) compared with that in the initial experiment (100:1) and incubated for 20 hours. Live/dead staining revealed that most bacteria within the macrophages remained viable over time (**Figure 4**). Collectively, these data demonstrate that *L. plantarum* can persist and proliferate within specialized macrophage compartments.

### Isolation and identification of *E. coli*

A total of 23 *E. coli* isolates were recovered from 5 out of 20 (25%) broiler farms. Among the recovered isolates, the predominant serotype was O126:K71 (80%). One of these isolates, confirmed to be nalidixic acid resistant, was selected and used as the challenge strain in the experimental infection model. The serotype O26:K60 represent (20%) of the isolated *E. coli*. These strains were primarily isolated from the heart, liver, gallbladder, lung, and air sacs (**Table 1**).

### Antimicrobial resistance profile isolation of APEC

**Figure 5:**
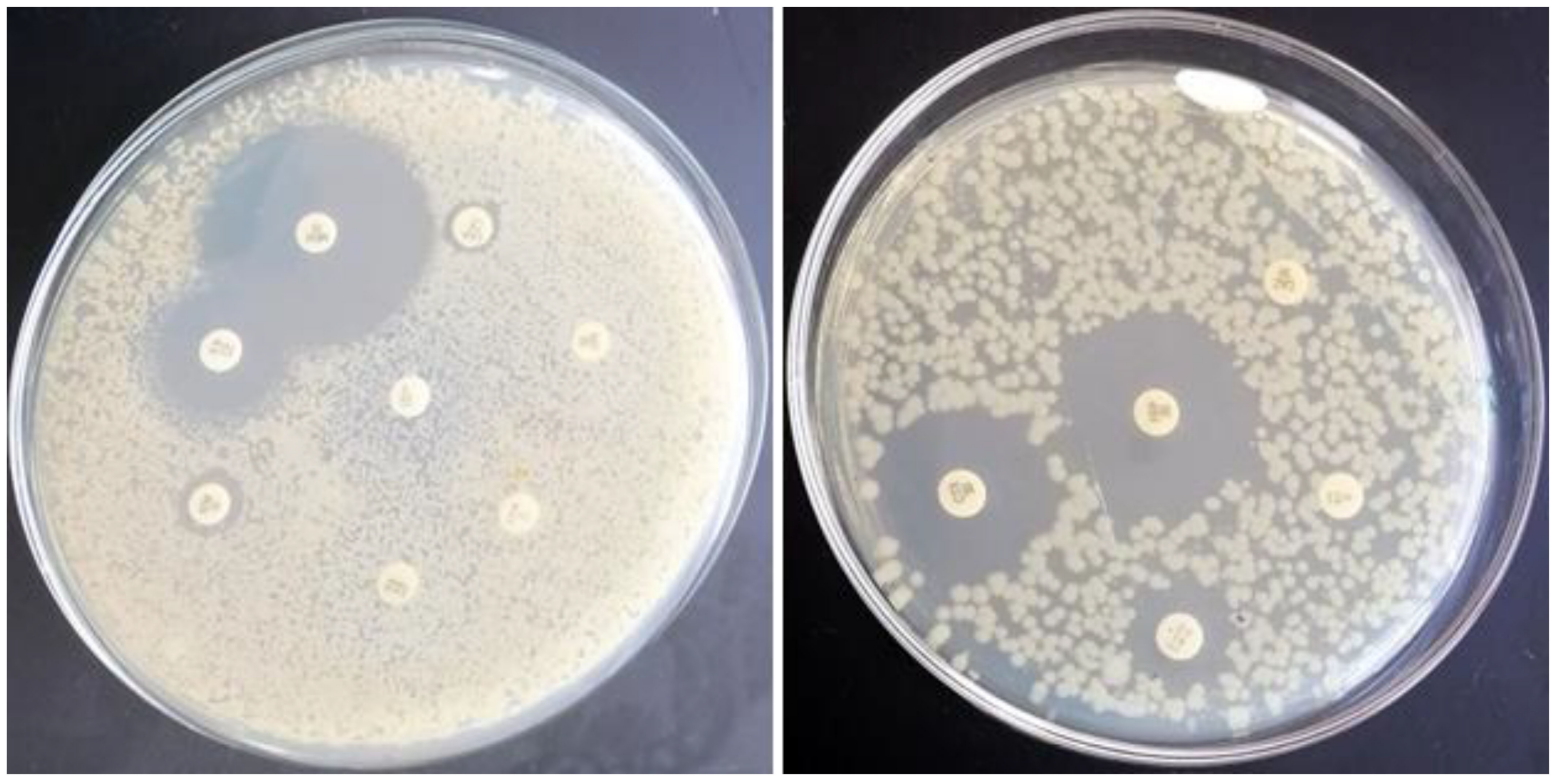
Phenotypic antimicrobial susceptibility of *E. coli* to antimicrobials that are frequently used in Egyptian broiler chicken farms. Representative results in which *E. coli* resists chloramphenicol (C), sulfamethoxazole & trimethoprim (STX), tetracycline (TE), cefoperazone (CFP), cefixime (CFM), aztreonam (ATM), streptomycin (S), and ceftazidime (CAZ), while sensitive meropenem (MEM), gentamycin (CN), cefotaxime (CTX), cefepime (FEP), and colistin sulfate (CT) are shown.

The *E. coli* isolates were tested against 14 commonly used antibiotics (**Table 1**). The overall resistance rate was 51.24%. The highest resistance was observed against cefixime and aztreonam (100%), followed by ceftazidime and cefotaxime (73.91%) and streptomycin (65.22%). In contrast, the isolates exhibited high sensitivity to imipenem and meropenem (100%), gentamicin (65.22%), and chloramphenicol (56.52%) (**Table 2**, **Figure 5**).

**Table 2:** Antimicrobial susceptibility of *Escherichia coli* isolates (n = 23) from broiler chickens, determined by the Kirby–Bauer disc diffusion method (26) and interpreted according to CLSI guidelines (27).

Antimicrobial agents	Disc concentration (µg)	Diameter of inhibition zone (mm)			Antimicrobial susceptibility profile		
		Resistant ≥mm	Intermediate mm	Sensitive ≤mm	Resistant	Intermediate	Sensitive
Chloramphenicol (C)	30	12	13-17	18	8	2	13
Sulphamethoxazole & trimethoprim (STX)	25	10	11-50	16	9	3	11
Tetracycline (TE)	10	14	15-18	19	13	2	8
Gentamycin (CN)	10	12	13-15	16	6	2	15
Streptomycin (S)	10	11	12-14	15	15	1	7
Colistin sulphate (CT)	10	10	11-13	14	9	8	6
Cefoperazone (CFP)	75	15	16-20	21	14	4	5
Cefepime (FEP)	30	18	19-24	25	11	4	8
Cefixime (CFM)	5	15	16-18	19	23	0	0
Aztreonam (ATM)	10	17	18-20	21	23	0	0
Ceftazidime (CAZ)	30	17	19-20	21	17	2	4
Cefotaxime (CTX)	30	22	23-25	26	17	1	5
Imipenem (IPM)	10	19	20-22	23	0	0	23
Meropenem (MEM)	10	19	20-22	23	0	0	23

Results show the number of isolates sensitive, intermediate, or resistant to each tested antibiotic.

### Clinical signs and postmortem lesions in the experimental infection

Chickens challenged with APEC (Group 3) developed respiratory signs within three days of inoculation, and 100% of birds showed postmortem lesions including airsacculitis, pericarditis, and hepatic congestion. In contrast, only 30% of birds in the *L. plantarum* + APEC group (Group 4) exhibited mild lesions, while no pathological changes were detected in the control (Group 1) or *L. plantarum*-only (Group 2) groups.”

### Growth performance and mortalities in the experimental infection

**Figure 6:**
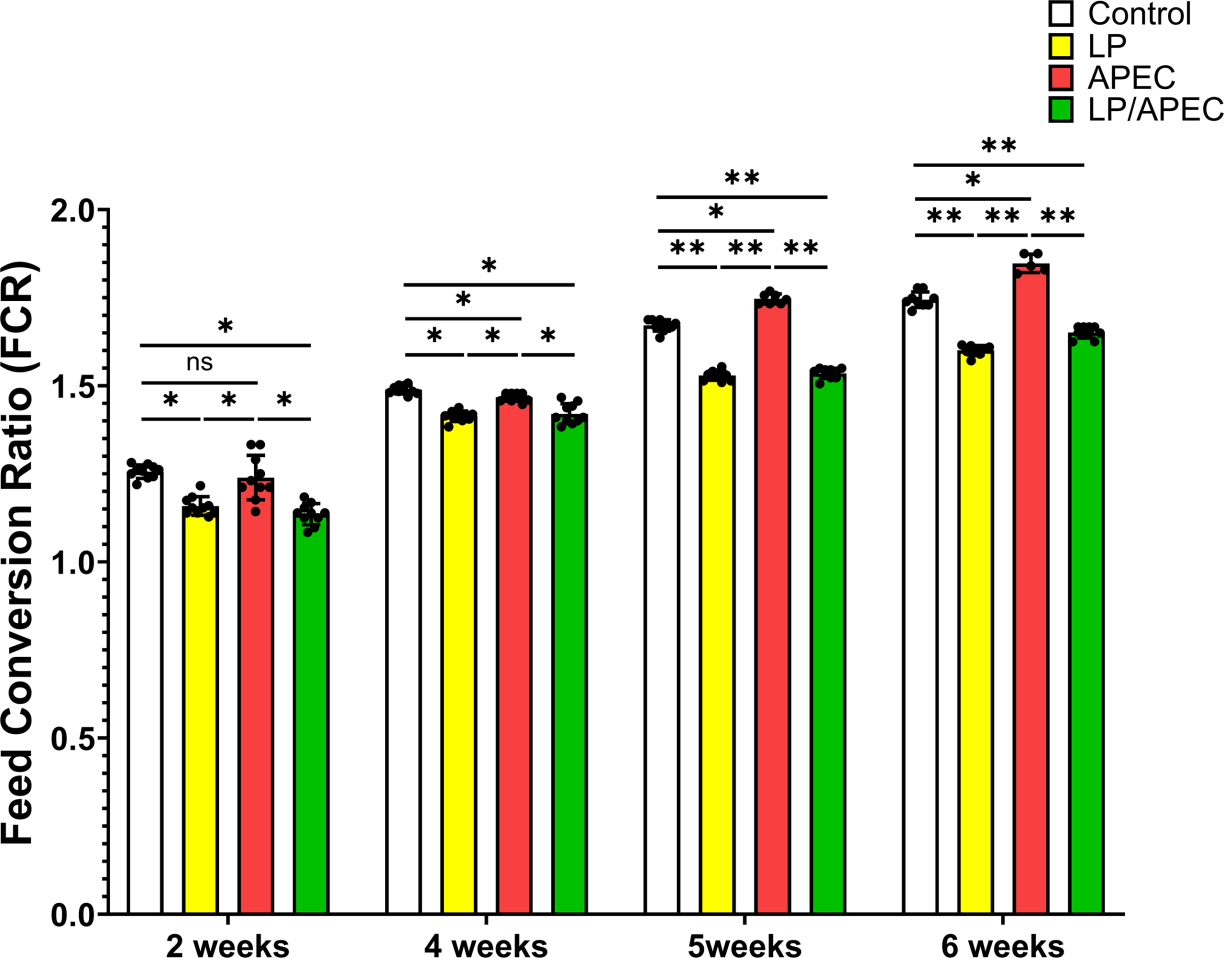
Effects of *Lactobacillus plantarum* dietary supplementation on feed conversion ratio (FCR) in control and APEC-challenged broiler chickens. Bars represent the mean ± standard deviation (n = 10 per treatment group). Statistical significance is indicated by asterisks (*p* < 0.05). *p < 0.05; **p < 0.01; ns, not significant.

**Figure 7:**
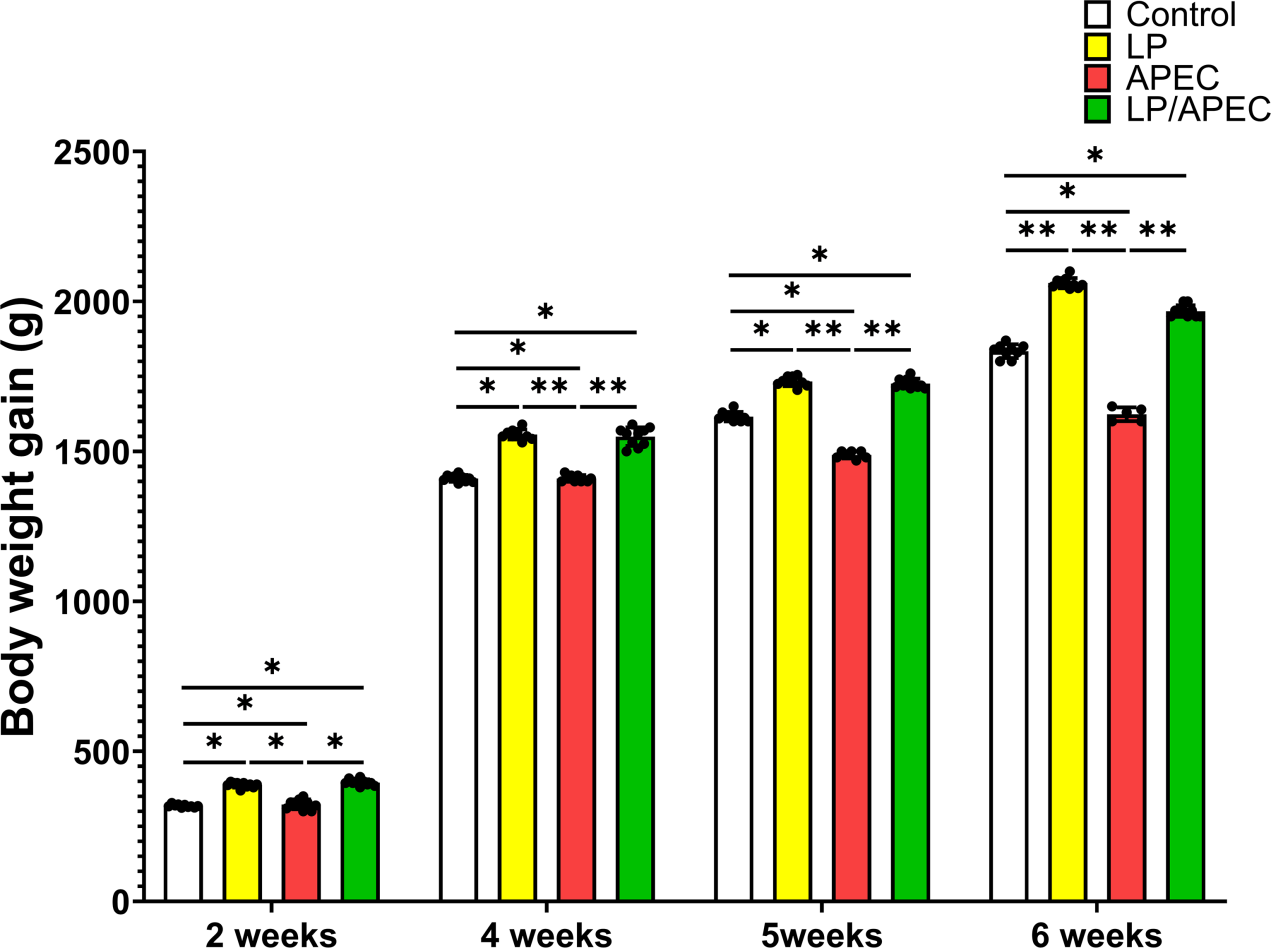
Effects of *Lactobacillus plantarum* (LP) supplementation on body weight gain in broiler chickens with or without *Escherichia coli* APEC challenge. Statistical significance is indicated by asterisks (*p* < 0.05). Body weight gain was significantly higher (*p* < 0.05) in the LP and LP + APEC groups compared with the control and APEC -challenged groups. The control group also differed significantly from the APEC -challenged group (*p* < 0.05). *p < 0.05; **p < 0.01.

Our results revealed that groups 2 and four presented statistically significant differences (*p* < 0.05) in FCR and increases in body weight gain compared with groups 1 and 3 throughout the entire experiment, especially in weeks 6 and 7 (**Figures 6**, **7**). On the other hand, after the challenge with APEC in group three, there was a significant reduction in the FCR and an increase in the body weight in the control group (G1) compared with those in the three APEC-infected groups.

Regarding the mortality rate, we recorded 0% mortality in both the control groups, which were fed a basal diet only (G1), and G2, which were fed a basal diet and were orally inoculated with *L. plantarum* However, there was significantly greater mortality (30%) in the APEC-challenged group than in the *L. plantarum* -treated and APEC-altered groups, with only 10% mortality (**Table 3**).

**Table 3:** Protective effects of *Lactobacillus plantarum* on mortality and recovery of *E. coli* after challenge of broiler chickens.

Group	Mortality	Isolation from liver
control	0	0
LB	0	0
*E. coli*	3 (30%)	10 (100%)
*Lactobacillus plantarum*/*E. coli*	1 (10%)	2 (20%)

### APEC cecal content bacterial count and isolation from liver

**Figure 8:**
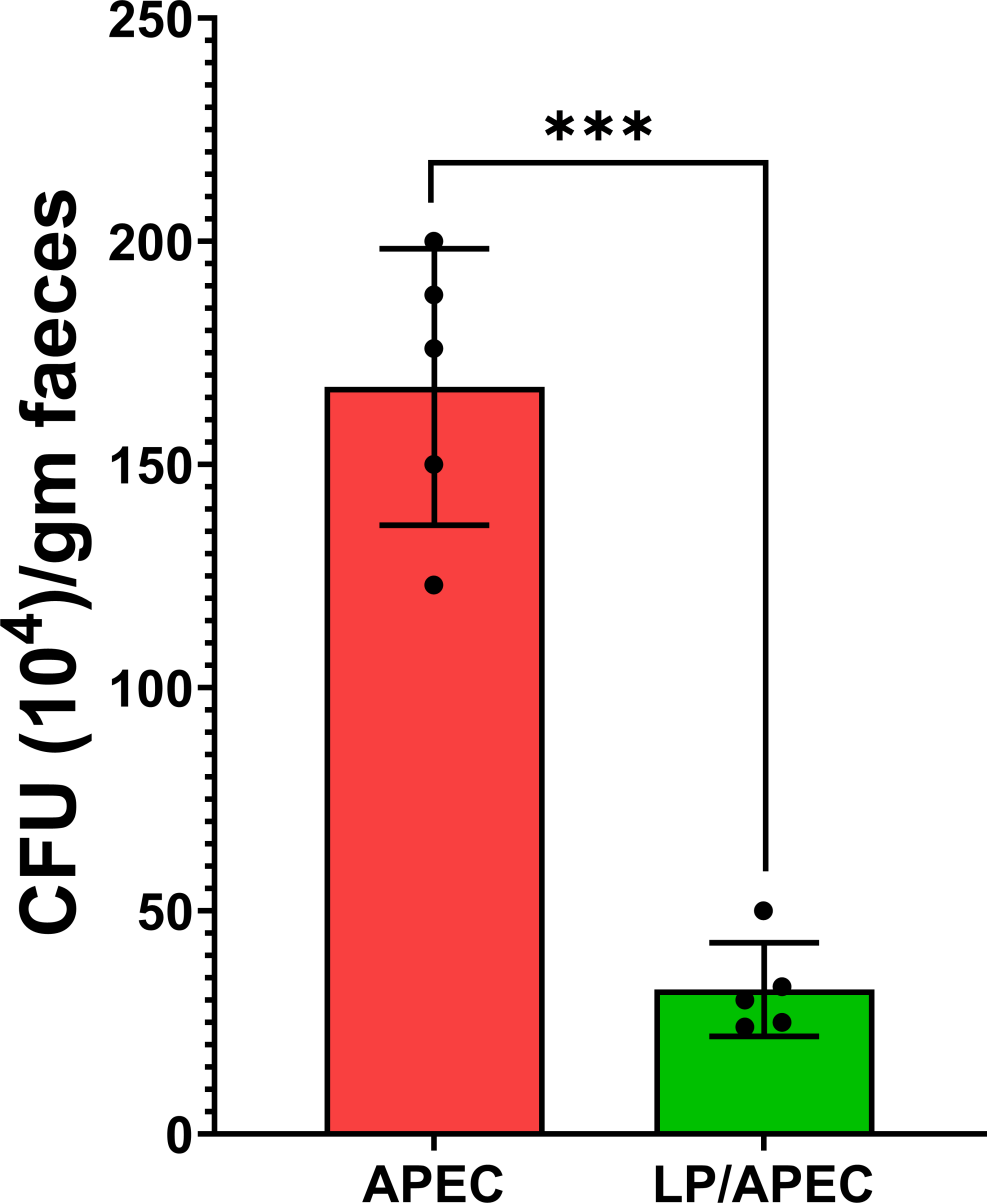
Effects of *Lactobacillus plantarum* (LP) supplementation on APEC cecal populations in broiler chickens. Bars represent the mean ± standard deviation. The APEC group was fed a basal diet for 28 days before challenge with APEC, whereas the LP/APEC group received a basal diet supplemented with LP for 28 days before the challenge. Cecal samples were collected on day 42. Colony-forming units (CFU) of APEC were significantly lower (p < 0.05) in the LP/APEC group compared with the APEC group. ***p < 0.001.

Oral supplementation with *L. plantarum* significantly (p < 0.01) reduced *E. coli* counts in the cecum compared with those in the untreated, APEC-challenged group (G3) (**Figure 8**). Additionally, *L. plantarum* significantly (p < 0.05) reduced APEC liver isolation rates from 100% in the untreated group to 20% in the treated group (**Table 3**).

### Serum Igy responses to treatment with *L. plantarum*

**Figure 9:**
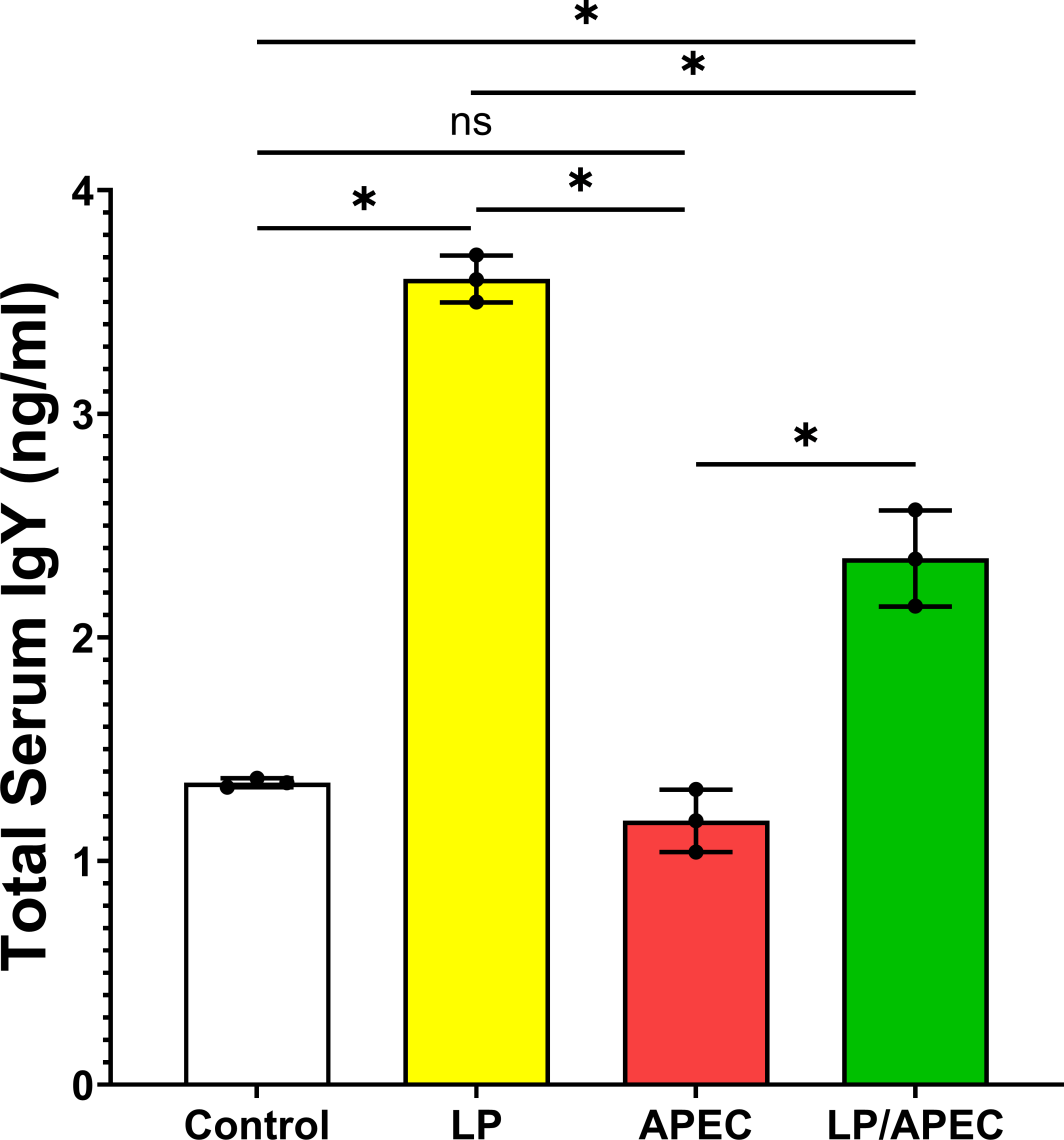
Serum total IgY antibody levels in broiler chickens two weeks after oral challenge with APEC. Group 1 received phosphate-buffered saline (PBS) as a control. Groups 2 and 3 were orally administered *Lactobacillus plantarum* (LP) from day 1 of age until the end of the experiment. Groups 3 and 4 were challenged with APEC on day 28. Serum samples were collected two weeks post-challenge. Bars represent the mean ± standard error of total IgY concentration (ng/mL). Statistical significance is indicated by asterisks (*p* < 0.05). No significant difference was observed between the control and APEC groups, while both LP and LP/APEC groups showed significantly higher IgY levels compared with the other two groups. The LP group exhibited a significantly higher IgY concentration than the LP/APEC group. *p < 0.05; ns, not significant.

To evaluate the protective effect of *L. plantarum* after challenge with APEC, the serum IgY content was measured via ELISA. Our results revealed significantly higher (*P<*0.01) levels of IgY than did the sera of the untreated control and APEC-infected nontreated groups (**Figure 9**). Additionally, the level of serum IgY in the *L. plantarum* treated group was significantly higher than the *L. plantarum* treated group and infected with *E. coli*.

## Discussion

The present work evaluated the potential of *Lactiplantibacillus plantarum* ATS1 to protect broiler chickens against avian pathogenic *E. coli* (APEC) infection. Our main objective was to assess both *in vitro* properties of the strain, such as adhesion and survival in host cells, and its *In Vivo* effects on growth, immune response, bacterial burden, and mortality after challenge. By linking laboratory findings with animal outcomes, this study provides insight into whether *L. plantarum* can be considered a practical probiotic intervention for reducing colibacillosis in poultry.”

The magnitude of antimicrobial resistance continues to rise, therefore, alternative strategies for disease management are urgently needed. Probiotics have emerged as promising candidates due to their ability to modulate the microbiota, enhance immune function (30–32). Several bacterial genera such as *Lactobacillus*, *Bacillus*, *Bifidobacterium*, *Enterococcus*, and *Escherichia* as well as certain yeasts, have demonstrated probiotic potential in poultry systems (33, 34).

Effective adhesion to the intestinal epithelium is a vital trait of probiotic microorganisms, as it facilitates colonization and enhances their interaction with the host. This adhesion is widely considered a primary indicator of probiotic efficacy (35, 36). A key mechanism by which probiotics exert protective effects is by competing with pathogenic microbes for attachment sites on the mucosal surface, thus limiting pathogen colonization a process known as competitive exclusion (14–16, 36, 37).

In this study, *L. plantarum* exhibited a strong and consistent attachment to Caco-2 cells, forming a distinct adherence pattern. Quantitative evaluation revealed an average of 5.65 bacterial cells adhering per epithelial cell, indicating strong colonization potential. These findings support previous observations that effective bacterial adhesion is central to reducing pathogen establishment and maintaining intestinal microbial balance (1, 38).

Beyond its interaction with epithelial cells, *L. plantarum* demonstrated the ability to survive and proliferate within murine macrophages an unusual trait for a non-pathogenic bacterium. The viability of *L. plantarum* inside macrophages, confirmed by live/dead staining, underscores the importance of this pathway in promoting intracellular persistence. This may, in turn, influence immune function, contributing to enhanced protection against infections.

The progressive increase in intracellular bacterial numbers and their localization within LAMP-1-positive compartments suggest that *L. plantarum* engages with host autophagy mechanisms. While such intracellular persistence is often exploited by pathogens to evade immune responses, our findings suggest that *L. plantarum* may utilize similar pathways in a beneficial context, potentially enhancing immune modulation rather than subverting host defenses.

Previous studies have reported that some probiotics can induce autophagy in epithelial cells through the secretion of bioactive, heat-stable, and acid-resistant proteins (39, 40). In our investigation, the intracellular survival of *L. plantarum*, implying that autophagy plays a supportive role in its persistence within immune cells. This interaction may represent a novel mechanism of probiotic-host communication, particularly relevant to poultry, where such intracellular probiotic behavior has not been widely studied.

The formation of *L. plantarum*-containing vacuoles (LpCVs) and their increasing bacterial load within macrophages from 3.05 ± 1.50 at 1 hour to 47 ± 5.58 at 20 hours post-infection (p<0.001) demonstrates not only survival but active replication. Co-localization with the lysosomal-associated membrane protein 1 (LAMP-1) confirms the involvement of the autophagy pathway. These results parallel previous findings in intracellular pathogens like *Salmonella* and *Listeria*, which recruit LAMP-1 to stabilize vacuolar membranes (41, 42). However, the presence of a probiotic strain within these structures highlights a novel, non-pathogenic use of this cellular machinery.

Our *In Vivo* data from broiler chickens supports the probiotic efficacy of *L. plantarum*. Oral supplementation led to significant improvements in growth performance, such as increased body weight gain and more favorable feed conversion ratios. In addition, probiotic-treated birds showed lower mortality rates and reduced APEC colonization in the liver and cecum, indicating a protective effect against bacterial infection. These findings confirm the protective role of *L. plantarum* against APEC infection, likely through modulation of the gut microbiota and immune stimulation. These results are consistent with previous reports on the benefits of probiotic supplementation in poultry (33, 34).

The development of septicemia, and respiratory distress, along with lesions such as airsacculitis, pericarditis, and lung and hepatic congestion in the APEC-infected group reflects the typical pathological picture of colibacillosis reported in broilers. The high frequency of these lesions in infected birds confirms the virulence of the O126:K71 strain. In contrast, probiotic supplementation markedly reduced lesion severity, with only mild changes detected in a minority of treated birds. This observation supports the protective role of *L. plantarum*, which may act through improving gut health and modulating immune responses. These outcomes are in agreement with earlier studies that linked probiotic use to reduced systemic dissemination and lesion formation in APEC-challenged flocks.”

The immunostimulatory effects of *L. plantarum* were further supported by elevated serum IgY levels in probiotic-treated groups (p<0.01). As IgY plays a central role in avian adaptive immunity, this increase suggests enhanced immune readiness, potentially driven by macrophage activation and cytokine signaling. The intracellular persistence of *L. plantarum* may serve as a continuous stimulus for such immune responses possibly through sustained macrophage engagement and autophagy-driven signaling pathways. These findings highlight a multifaceted mode of action involving both direct antimicrobial activity and immunomodulation.

Although higher serum IgY concentrations in birds receiving probiotics indicate enhanced humoral immunity, this parameter alone does not capture the full complexity of the immune response. Future studies should expand the immunological assessment to include factors such as mucosal IgA, a range of pro- and anti-inflammatory cytokines, and T-cell activity to better characterize both systemic and localized immune effects. Moreover, while APEC colonization in the cecum and liver dissemination was measured, the complex interactions among APEC, *L. plantarum*, and the resident gut microbiota remain unexplored. Investigating the competitive interactions between pathogens and commensal bacteria, as well as how *L. plantarum* influences microbiota composition and gut barrier function, should be addressed in future work using metagenomic or microbial community analysis techniques.

The prevalence of APEC among the sampled farms was 25%, with the dominant serotypes being O126:K71 (80%) and O26:K60 (20%). The clinical presentation in infected birds respiratory signs, septicemia and the associated gross lesions, such as air sacculitis and pericarditis, were consistent with earlier studies (43, 44). The observed mortality rate of 3.47% ± 0.93 further reflects the pathogenic impact of APEC on broiler production.

The study also provides updated insights into antimicrobial resistance patterns among APEC isolates. A high resistance rate (51.24%) was detected, with 100% resistance to cefixime and aztreonam, and over 70% resistance to ceftazidime and cefotaxime, in addition to significant resistance to streptomycin (65.22%). These resistance patterns reflect global concerns and highlight the risks associated with excessive and unregulated use of antimicrobials in poultry farming (7, 34).

This study fills a significant gap in current knowledge by elucidating the cellular mechanisms underlying the probiotic activity of *L. plantarum* against multidrug-resistant APEC. While many prior studies have demonstrated the general benefits of probiotics in poultry, such as enhancing performance and gut health, the intracellular behavior of probiotic strains within immune cells has been largely overlooked. Our findings reveal that *L. plantarum* not only adheres to epithelial cells but also survives and proliferates within murine macrophages, localizing to LAMP-1-positive vacuoles, indicative of autophagy involvement. This intracellular persistence suggests a previously unrecognized immunomodulatory mechanism. Moreover, the study uniquely combines field data on APEC antimicrobial resistance with functional *in vitro* and *in vivo* analyses, making it among the first to provide a comprehensive assessment of *L. plantarum*’s role as a probiotic alternative to antibiotics in broiler chickens.

## Limitations and recommendations for future research

Several limitations of the present study should be acknowledged to guide future research on probiotic–pathogen–host interactions. First, histopathological evaluation of the liver and intestine after APEC challenge was not performed. Although infection was confirmed by re-isolation from liver tissue, microscopic assessment would have provided additional information on tissue damage, inflammatory cell infiltration, and structural alterations. Likewise, examining the expression of tight junction proteins and adhesion molecules by qRT-PCR could have yielded molecular evidence of probiotic protection at intestinal and hepatic levels.

Second, the study did not examine gut microbiota composition, which could have clarified how probiotic supplementation affects microbial balance and intestinal health. Future investigations should include microbiome profiling to better understand host–microbe interactions and the mechanisms underlying probiotic action.

Third, the probiotic strain tested was a partially characterized field isolate (*L. plantarum* ATS1, GenBank accession no. PV478081.1) without comparison to a reference strain. While ATS1 showed promising probiotic activity *in vitro* and *in vivo*, the absence of a benchmark strain (e.g., *L. plantarum* ATCC 8014) limits the generalizability of the findings. Similarly, the challenge model employed only one pathogenic *E. coli* serotype (O126:K71), which restricts broader conclusions. Future studies should include both reference probiotic strains and standardized APEC isolates to validate strain-specific effects.

Fourth, the biofilm-forming ability of APEC O126:K71 was not investigated, even though biofilms are important for persistence and resistance. Future work should explore whether *L. plantarum* can prevent biofilm formation or disrupt mature biofilms, thereby enhancing its protective role against persistent infections.

Finally, the strain was administered without encapsulation, yet still improved growth, immunity, and reduced colonization. This suggests that viable cells survived gastrointestinal passage; however, acidic and enzymatic conditions are known to reduce probiotic viability in practice. Encapsulation methods using lipid or polymer carriers may improve stability, survival, and targeted delivery, increasing the consistency of probiotic effects under field conditions.

## Conclusion

Avian pathogenic *E. coli* was isolated from diseased broilers with diverse serotypes and a high rate of antimicrobial resistance, confirming its ongoing threat to poultry production. *In vivo* supplementation with *Lactiplantibacillus plantarum* ATS1 improved growth performance, enhanced IgY response, reduced tissue colonization, and lowered mortality following challenge. These results highlight both the burden of multidrug-resistant APEC and the potential of *L. plantarum* as a probiotic approach for controlling colibacillosis.

## Data Availability

The datasets presented in this study can be found in online repositories. The names of the repository/repositories and accession number(s) can be found below: https://www.ncbi.nlm.nih.gov/genbank/, 1.https://www.ncbi.nlm.nih.gov/nuccore/PV478081.1/.
